# *QuickStats*: Average Age at Death*^,^^†^ by Race/Hispanic Origin and Sex — National Vital Statistics System, United States, 2017

**DOI:** 10.15585/mmwr.mm6831a4

**Published:** 2019-08-09

**Figure:**
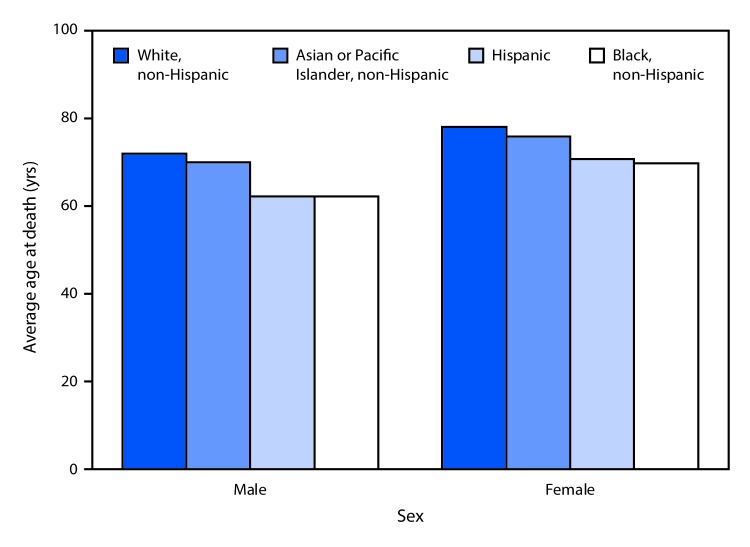
In 2017, in the United States, the average age at death among males was highest for non-Hispanic whites (72.0 years), followed by non-Hispanic Asians or Pacific Islanders (70.0), Hispanics (62.2), and non-Hispanic blacks (62.1). Among females, the average age at death was highest for non-Hispanic whites (78.1 years), followed by non-Hispanic Asians or Pacific Islanders (75.8), Hispanics (70.7), and non-Hispanic blacks (69.7).

**Source:** National Vital Statistics System. Underlying cause of death data, 1999–2017. https://wonder.cdc.gov/ucd-icd10.html.

